# Metropolitan Hospital Sunday Fund

**Published:** 1896-06-13

**Authors:** 


### THE INSTITUTIONAL WORKSHOP.

### METROPOLITAN HOSPITAL SUNDAY FUND.

### PRESENTATION BY THE PRINCE OF WALES TO MR. BURDETT.

A special meeting of the Council of the Metropolitan Hospital Sunday Fund was held on the 8th inst., when H.R.H. the Prince of Wales, the Vice-Patron of the Fund, who was accompanied by H.R.H. the Duke of York, presented the Council's album to Mr. Burdett in recognition of his exertions last year in raising the amount collected by the Fund to £60,000. Colonel A. Stanley Clark was in attendance on the Prince of Wales, and Sir Charles Cust on the Duke of York.

The ceremony took place in the old Ball Room at the Mansion House, the Lord Mayor, the President of the Fund, in the chair. With the Lord Mayor were the Lady Mayoress, the Sheriffs, and several officers of the Corporation; while amongst those present were Earl Stanhope, Lord Knutsford, Lord Frederick Fitzroy, Sir James Paget, Bart., F.R.S., Sir Wm. Broadbent, Sir Richmond Cotton, Mr. R. B. Martin, M.P., Sir Sydney Waterlow, Bart., Sir Savile Crossley. Bart., Sir George Bruce, the Bishop of Stepney, Archdeacon Sinclair, Cannon Fleming, Canon Ingram, Canon McCormick, the Chief Rabbi, the Greek Archimandrite, the Rev. Dr. Barlow, the Rev. Dr. McLeod, the Rev. Dr. Rigg, the Revs. S. B. Burnaby, W. T. Du Boulay, W. H. Harwood, J.F. Kitto, C. J. Ridgeway, David Anderson, M. R. Neligan, and Russell Wakefield, Colonel F. Haygarth, Captain James Cundy, Captain Arthur Palliser, Messrs. W. H. Burns, J. H. Buxton, F. C. Carr-Gomm, A. L. Cohen, Dr. C. J. Hare, Messrs. H. Hoskier, A. G. Sandeman, W. R. Tidd, A. Willett, J. Astley Bloxam, T. Wakley, H. N. Custance, and R. A. Owthwaite.

The LORD MAYOR explained that His Royal Highness had that day honoured them with his presence—showing thereby his interest as vice-patron in a society which had done so much good—in order to assist in the ceremony of presenting one of their number with some slight recognition of the services he had rendered to the Hospital Sunday Fund. (Applause.) He then called upon Sir Sydney Waterlow to read the resolution passed by the Council.

Sir SYDNEY WATERLOW read the following resolution:—

That the thanks of the Council be given to Henry C. Burdett, Esq., for the great interest he has taken in the Metropolitan Hospital Sunday Fund for many years past, for the benefit that the Fund has derived from his advocacy of its claims in THE HOSPITAL newspaper and by other means, but especially for his strenuous exertions and personal influence this year in obtaining subscriptions raising the Fund to the unprecedented sum of over £60,000.

Continuing, he said: The Lord Mayor has been kind enough to summon the members of the Hospital Sunday Fund to-day for the purpose, as the resolution tells you, of presenting to our friend, Mr. Henry C. Burdett, this magnificent album. (Applause.) The book contains photographs of Her Majesty the Queen, our patron; His Royal Highness the Prince of Wales, vice-patron; the President, the Vice-Presidents, and the members of the Council. It has been prepared by order of the Council, as they desire to show their friend, Mr. Burdett, their great appreciation of the services he has rendered to the Fund during the last ten or twelve years, not merely as an energetic member of the Council, not only as an advocate of the Fund in his excellent medical journal THE HOSPITAL, but in many other ways, for Mr. Burdett has continually laboured to bring the Metropolitan Hospital Sunday Fund before the public and to increase the amount annually collected. (Hear, hear.) But, sir, these efforts were especially accentuated during last year. I think Mr. Burdett felt—as many of us did, but he especially felt—that the sum which we had been in the habit of collecting annually was not such a sum as should be collected in such a very large and wealthy city as London. By means of the good influence he exercises over many of the wealthiest merchants in the City, he added the sum of £15,000 to the amount collected by the Hospital Sunday Fund in 1895, raising the collection to £60,861, or one-third more than had been collected in any previous year, and more than twice the amount which was collected on the first occasion that Hospital Sunday was held in London. (Applause.) The total receipts of the Fund during the twenty-three years ended in 1895 amounted to £828,697. The annual receipts have increased from year to year, and the number of congregations contributing to the Fund have increased from 1,072 in the first year to 1,871 last year. In other words, the numbers have nearly doubled, thus showing the increased popularity of the Fund, and the increased confidence the public have in it. In 1894 we received a legacy of £45,346 2¾ per cent. Consols, and if that sum were capitalised and added to the amount collected, we should have raised, including the collection to be made this year, £1,000,000. It has been said—and said, I think, with truth—that this million of money collected in twenty-four years is not a large sum for a city like London to have raised, especially when we bear in mind that the profits made in the City proper, that small area controlled by the Lord Mayor and the Corporation, exceed £1,000,000 a week (last year more than £52,000,000 was returned as profits). With the knowledge that this is the income we do look forward to maintain the £60,000 a year standard at least, and not to let the Fund go back. (Hear, hear.) My own impression, after working at this Fund since its inauguration, is that we ought not to be satisfied until the metropolis raises £100,000 a year for the relief of the sick poor. (Applause.)

#### THE PRESENTATION BY THE PRINCE.

The Prince of WALES, who was greeted with loud applause, said: My Lord Mayor, ladies, and gentlemen, I feel it a great compliment and a great honour to have been asked to present to Mr. Burdett this album which has been accorded to him by the council of the Hospital Sunday Fund in recognition of the successful efforts made by him last year to raise the Fund to the unprecedented total of £60,000. In doing so I feel that I am representing all classes of the community, and I am sure there is no one who for his practical philanthropy, if I may use the term, has more richly deserved the appreciation and the thanks of his contemporaries. (Applause.) You are aware, ladies and gentlemen, that Mr. Burdett studied for the medical profession at Guy's Hospital, but since 1880 he has filled another and most important post, that of secretary to the Share and Loan Department of the Stock Exchange. He has, as you are aware, always taken the deepest interest in the charitable institutions of his country, and he has written much on the subject. Recently he published a work entitled "Hospitals and Asylums of the World," which the *Times* very justly described as his "monumental work," giving the origin, history, management, administration, and plans of the various medical charities of known existence. This book is a most valuable encyclopædia which everybody ought to possess. Mr. Burdett organised a system of training nurses according to modern ideas, which has been most successful. In six years he raised the income of the Dreadnought Hospital from £7,000 to £13,000 a year. He established the Home Hospitals Association for paying patients in Fitzroy Square, raising a sum of £26,000 for that object. He has also founded an institution in which the Princess and myself take a deep interest—the National Pension Fund for Nurses. (Applause.) Mainly through his efforts the endowment of this Fund has reached £60,000, and last year's report shows that the total investments amount to nearly £300,000. (Applause.) During a single year he has had £37,000 sent to him at his private house for charitable purposes, and in 1895 £50,000 was placed in his hands in one month for distribution amongst charitable institutions. From the commencement he has been interested in the work of the Hospital Sunday Fund. He has aided it by indefatigable work on the Council, and also by his pen, his purse, and his City influence. (Applause.) In the first year of the fund the collections amounted to £27,700; last year it was over £60,000, towards which he raised £15,000. What Mr. Burdett has done has justly been recognised by the Council, headed by the Lord Mayor, in that special resolution which is enclosed in the album. (Applause.) I know Mr. Burdett's name is so well known to all of you, especially in this great city of London, that little is left for me to say, but I venture to say this—and I believe all of you will endorse it—that for what Mr. Burdett has done for the great charitable institutions of this country his name will go down to posterity as it is at present—a household word. (Loud applause.) On behalf of the Council, I have the pleasure to present this album to you. (Renewed applause.)

Mr. Burdett's Acknowledgment.

Mr. Henry C. Burdett returned thanks as follows:—May it please your Royal Highnesses, my Lord Mayor, my lords, ladies, and gentlemen,—To adequately return thanks for this spontaneous and unanimous token of goodwill and appreciation for humble endeavours made under a full sense of responsibility in the cause of the sick and suffering of this vast metropolis, is a pleasant but by no means an easy matter. That I should receive it at the hands of your Royal Highness adds both to the value and the interest of the gift. (Applause.) In accepting with grateful heart this beautiful album, I regard myself as the too fortunate representative of the many zealous friends of the hospitals through whose co-operation last year's record of £60,000 was secured, a sum of which the London hospitals stand in need every year at the hands of the Sunday Fund. Personally, I regard it as a high privilege to be able to give of myself and of my limited time to a cause which is very near my heart. (Hear, hear.) It was through personal suffering that I first became fully alive to the distress represented by the occupants of a hospital bed, which twelve years' residence within hospital walls had failed to teach me so effectually. I cannot forget that there are upwards of 9,000 hospital beds in London to-day. What an immensity of suffering they must collectively represent! Truly, all that Londoners can do, both by personal service and by monetary assistance, is not too much for the needs of the multitude of the sick poor which the metropolis contains. Last year the gifts of the laity were large and spontaneous; this year the congregational collections may be expected to reach a larger total, for the clergy, to whom the Lord Mayor has made special appeal, promise to enter more heartily than ever into the work. May I here say that I personally wish to thank the present Lord Mayor and also his predecessor for their great kindness to me. Your Royal Highness's presence here to-day, the first occasion on which you have attended a meeting of the Hospital Sunday Fund since your acceptance of the office of vice-patron early this year, cannot fail to encourage old friends of the hospitals, and to enlist new ones on their behalf. Speaking personally, your Royal Highness's invariable readiness to help the hospitals of this country has often inspired others like myself to fresh efforts—(applause)—and the gift which I have received to-day, this album containing the photograph and autograph of every member of the Council, including those of Her Majesty the Queen as patron and of your Royal Highness, will ever be regarded by me as one of my most valued possessions and by my family as a precious heirloom. When working days are over I shall turn with gladdened eyes towards this album, rich with memories of those kindly friends and of you, my colleagues, united to me for so long a period by bonds of sympathy which are the outcome of mutual endeavours for the alleviation of the sufferings of mankind. Your Royal Highnesses, my Lord Mayor, ladies, and gentlemen, I thank you all from the bottom of my heart. (Applause.)

The proceedings then terminated.

Description of the Album.

The album, which is the work of Albert Barker (Limited), 5, New Bond Street, measures 24 in. by 18 in., and is bound in green crocodile leather with richly-gilt ormolu corners and clasp. In the centre of the cover are the crest, coat of arms, and motto of the recipient richly carved and pierced in ormolu. Internally it is lined with white moiré silk. It contains altogether 113 photographs, all except two being those of the Council of the Fund, each bearing the signature of the giver. The place of honour is accorded to the portrait of Her Majesty, who is Patron of the Fund, and then follows that of the Prince of Wales as Vice-Patron. The album opens with the following inscription:—

Metropolitan Hospital Sunday Fund.—Patron, Her Most Gracious Majesty the Queen; vice-patron, His Royal Highness the Prince of Wales, K.G.—Presented to Henry C. Burdett, Esq., on behalf of the Patron, President, and Council, by H.R.H. the Prince of Wales, at a special meeting held at the Mansion House, London, on Monday, June 8th, 1896, as a mark of their appreciation of the great interest he has taken in the Metropolitan Hospital Sunday Fund for many years past; of the benefit which the Fund has derived from his advocacy of its claims in The Hospital newspaper, and by other means; but especially by his strenuous exertions and personal influence in obtaining subscriptions, raising the Fund to the unprecedented sum of £60,000 during the past year.—On behalf of the Council, Sir Walter Wilkin, Lord Mayor; Sir Sydney H. Waterlow, Bart., Sir Edward Hay Currie, Richard B. Martin, M.P., Hon. Secretaries; Henry N. Custance, Secretary.

The album is fixed to a carved mahogany easel, to obviate any possibility of damage through frequent opening, with a new patent spring action specially invented.

By request the album will be on view at the makers, Albert Barker (Limited), 5, New Bond Street, W., until the end of the present month.

